# The Role of TRPM4 in Cardiac Electrophysiology and Arrhythmogenesis

**DOI:** 10.3390/ijms241411798

**Published:** 2023-07-22

**Authors:** Yaopeng Hu, Jiehui Cang, Keizo Hiraishi, Takayuki Fujita, Ryuji Inoue

**Affiliations:** Department of Physiology, Fukuoka University School of Medicine, Fukuoka 814-0180, Japan

**Keywords:** TRPM4 channel, Ca^2+^ homeostasis, cardiac electrophysiology, arrhythmogenesis

## Abstract

The transient receptor potential melastatin 4 (TRPM4) channel is a non-selective cation channel that activates in response to increased intracellular Ca^2+^ levels but does not allow Ca^2+^ to pass through directly. It plays a crucial role in regulating diverse cellular functions associated with intracellular Ca^2+^ homeostasis/dynamics. TRPM4 is widely expressed in the heart and is involved in various physiological and pathological processes therein. Specifically, it has a significant impact on the electrical activity of cardiomyocytes by depolarizing the membrane, presumably via Na^+^ loading. The TRPM4 channel likely contributes to the development of cardiac arrhythmias associated with specific genetic backgrounds and cardiac remodeling. This short review aims to overview what is known so far about the TRPM4 channel in cardiac electrophysiology and arrhythmogenesis, highlighting its potential as a novel therapeutic target to effectively prevent and treat cardiac arrhythmias.

## 1. Introduction

The transient receptor potential melastatin 4 (TRPM4) channel is a Ca^2+^-activated non-selective cationic (NSca) channel, with a unitary conductance of approximately 20pS [1]. The TRPM4 channel protein is ubiquitously expressed in many kinds of cells, where it participates in intracellular Ca^2+^ homeostasis and modify excitability by influencing the membrane potential. Among the widespread expressed tissues, TRPM4 protein is also abundant in the heart [2]. Several lines of evidence suggest that TRPM4 channels may be involved in arrhythmogenicity in the heart, with both inherited and acquired traits. For example, a gain-of-function mutation (GOF) on its distal N-terminal domain has been reported to produce degenerative changes in the cardiac Purkinje system, and has been identified in a few pedigrees of Jewish and French families that manifest progressive conduction blocks and associated sudden death [3]. In spontaneously hypertensive rats (SHRs), long-term pressure overload produces hypertrophic changes in the heart accompanied by upregulation of TRPM4 channel proteins and their excessive activities [4]. These changes are further associated with the prolongation of QT interval in electrocardiogram, which is a risk factor for lethal arrhythmias. In murine hearts exposed to acute anoxic insults, early afterdepolarization (EAD)-like oscillations in the repolarization phase of action potential (AP) occur, which are selectively inhibited by a TRPM4 channel blocker, 9-phenathrol [5]. All these observations are consistent with the idea that the TRPM4 channel may play non-trivial roles in arrhythmogenesis.

This review succinctly summarizes the characteristic properties of the TRPM4 channel and discusses its implications in cardiac arrhythmogenicity.

## 2. Biophysical Properties of TRPM4

The TRPM4 channel has a large cytosolic domain. Until 2017, the structure of the TRPM4 channel remained unknown. However, since then, several TRPM4 structures have been resolved in different ligand-bound states using single-particle cryo-electron microscopy (EM). All of these structures were determined for the closed state of the TRPM4 channel, while its open-state conformation is still elusive [6,7,8,9].

Like many other TRPM subfamily members, TRPM4 has an N-terminal TRPM homology region (MHR) domain, a transmembrane domain (TMD) that includes six transmembrane helices, a C-terminal coiled-coil domain, a TRP helix, and a C-terminal domain (CTD). The ion-conducting pore domain is formed by the transmembrane S5 and S6 helices and surrounded by the S1–S4 domain. These two crucial domains are connected through the S4–S5 linker, forming a domain-swapped conformation that may play an important role in the gating of the TRPM4 channel [6]. The S1–S4 domain of the TRPM4 channel contains binding sites for Ca^2+^ and other ligands [8], as well as a voltage sensor-like domain (VSLD) reminiscent of the counterpart of classical voltage-gated ion channels [10]. However, the VSLD of TRP channels shows a small number of gating charges; in TRPM4, it is only −0.7e [11], which may be mediated by polar residues in and around the putative fourth transmembrane domain. Nonetheless, this charge appears important for the channel’s voltage dependence, though more compelling evidence such as from mutagenesis studies is necessary.

While not primary itself, the membrane depolarization strongly modulates the TRPM4 channel’s activity; hence, the TRPM4 channel shows a prominent dependence on the physiological range of membrane potentials, once activated by an increase in the intracellular Ca^2+^ level ([Ca^2+^]_i_). This is similar to other types of Ca^2+^-activated channels, such as BK_Ca_ [12]. The voltage dependence of the TRPM4 channel is an important feature in cardiac tissues, where it affects the membrane potential over a wide range. Incorporation of this property into simulation models shows that the TRPM4 channel is most prominently activated at the late repolarization phase of action potentials (APs) and when the resting [Ca^2+^]_i_ is high (in the high submicromolar and micromolar ranges), and is substantially active even near the maximum diastolic potential (MDP) [13]. Therefore, TRPM4 may contribute primarily to the prolongation of APs and, to a lesser extent, to the generation of abnormal diastolic depolarizations that can occur after complete termination of APs. While TRPM4 may not be a major contributor to pace-making potentials, it can still play a role in modulating cardiac excitability and arrhythmogenesis.

The Ca^2+^ binding sites of the TRPM4 channel are thought to exist within a hydrophilic pocket (Glu^828^, Gln^831^, Asn^865^) of the cytosolic parts of S2 and S3 transmembrane helices. Beneath these binding sites is a very narrow space that allows cytosolic Ca^2+^ to pass through, between the S2-S3 linker and the TRP domain. One of the only two positively charged amino acid residues of the S4 domain (Arg^905^) is located just above the S2–S3 linker [6]. This positioning may facilitate coordination and priming effects for voltage-dependent opening. It has also been reported that phosphatidylinositol bisphosphate (PIP_2_) binding as well as calmodulin interaction are involved in maintaining and enhancing the Ca^2+^ sensitivity of the TRPM4 channel [14]. Therefore, understanding and evaluating the Ca^2+^ sensitivity of the TRPM4 channel at physiological or submicromolar Ca^2+^ levels is very important in elucidating its exact roles in arrhythmogenicity [13].

## 3. Physiological Roles of TRPM4 in the Heart

(1) Contribution of TRPM4 to sinoatrial (SA) nodal and other cardiac automaticity

In the SA node (SAN), intracellular Ca^2+^ oscillations generated by the Ca^2+^ clock play a critical role in regulating cardiac automaticity. During the diastolic depolarization phase, the voltage-dependent Cav1.3 L-type Ca^2+^ channels (LTCC) activate, then Ca^2+^ influx into the SAN cell triggers Ca^2+^ release from the sarcoplasmic reticulum (SR) via the ryanodine receptors (RyR) [15]. A subsequent rise in [Ca^2+^]_i_ activates the Na^+^-Ca^2+^ exchange (NCX), which in turn extrudes Ca^2+^ from the cell in exchange for Na^+^. This generates an inward Na^+^ current that contributes to the depolarization of the membrane potential and increases the rate of pace-making diastolic depolarization (DD) [16].

TRPM4 channels possibly contribute to the regulation of cardiac automaticity by modulating both the Ca^2+^ clock and membrane clock in the SAN [17]. It has been suggested that the TRPM4 channel may contribute to the inward Na^+^ current during the DD phase [18]. The TRPM4 channel may also have additional effects on the cardiac automaticity, such as modulating the inward driving force for Ca^2+^ and [Ca^2+^]_i_ [19]. It has been reported that inhibition of TRPM4 channels by 9-phenanthrol reduces the heart rate in mice, rats and rabbits, suggesting that the TRPM4 channel may act as an accelerator of DD when the heart rate is decreased, to avoid bradycardia [18,20].

Overall, the TRPM4 channel plays a critical role in regulating cardiac automaticity by modulating the DD slope and [Ca^2+^]_i_ in the SAN, and its precise contribution to the inward Na^+^ current and the DD phase is still an ongoing focus of investigation.

(2) Role of TRPM4 channel in the atrial myocardium

The TRPM4 channel has been shown to be involved in atrial electrophysiology [21]. It is expressed in mice, rats, and human atrial cardiomyocytes [21,22,23]. The electrophysiological function of the TRPM4 channel in atrial APs was evaluated in isolated atrial cardiomyocytes by using TRPM4 knockout (*Trpm4 KO*) mice and a selective inhibitor, 9-phenanthrol. Inhibition of the TRPM4 channel shortened the AP duration of isolated atrial cardiomyocytes compared to those in wild-type (WT), but not knockout animals. The duration of atrial APs was also shorter in *Trpm4 KO* compared to WT animals [21].

The TRPM4 channel is reported to be responsive to shear stress induced by IP_3_ receptor-mediated Ca^2+^ releases in rat atrial cardiomyocytes [23]. TRPM4 has also been implicated in aldosterone-induced atrial arrhythmias [24]. In the same study, disorganization of connexin-43 (Cx43) in atria was observed in *Trpm4 KO* mice, more than in WT mice. This phenomenon may be involved in the occurrence of atrial electrical disturbances. Additionally, TRPM4 channel may contribute to the growth of human and mice atrial fibroblasts; both expression and functional currents of TRPM4 has been shown to increase under cultured conditions [25]. Presumably, the TRPM4 channel may be engaged in some way commit to the process of atrial fibrosis [25,26].

In summary, the physiological role of TRPM4 in the atrial myocardium appears to be complex and context-dependent, with a variety of consequences observed under different conditions. It may thus be reasonable to speculate a nontrivial role(s) of TRPM4 in the pathogenesis of atrial fibrillation (AF), through alterations of both atrial electrophysiology and remodeling.

(3) Role of TRPM4 channel in the ventricular myocardium and Purkinje conduction system

The physiological role of the TRPM4 channel in the ventricular myocardium remains controversial. Discrepancies in the contribution of the TRPM4 channel between the atrium and ventricle has been reported, following comparisons of both the transcript level and functional current. Although highly detected in the atrium, the TRPM4 channel is much less expressed in the ventricular myocardium [27]. The definitive contribution of the TRPM4 channel to the AP duration of canine ventricular cardiomyocytes has been confirmed in the latest study by using a potent and highly selective inhibitor, 4-chloro-2-[[2-(2-chlorophenoxy) acetyl] amino] benzoic acid (CBA) [28]. In contrast, the *Trpm4* mRNA detection and functional Ca^2+^-activated nonselective cation current were greatly enhanced in the ventricular cardiomyocytes of SHRs compared to those in the Wistar-Kyoto (WKY) rat [4]. The same study observed QT prolongation in SHRs, which is accompanied by an increased TRPM4 channel activity. Furthermore, the TRPM4 channel was involved in the positive inotropic effect of β-adrenergic stimulation in the ventricular myocardium [29].

The network of terminal Purkinje fibers (PFs) carries electrical impulses to the ventricular myocardium, playing a central role in the excitation-contraction cycle of the ventricle. PFs demonstrate a unique electrophysiology with complicated intracellular Ca^2+^ cycling [30]. The TRPM4 channel is most abundantly expressed in PFs, compared to the other human heart tissues [31], suggesting its significant contribution to the electrical properties of PFs [32]. The propagation failure of PFs caused by TRPM4 channel overexpression has been observed in silico [33]. More intriguingly, optical mapping of ectopic activation induced by mechanical stimulation in PFs demonstrated a clear link with the activation of TRPM4 channels [34]. The involvement of the TRPM4 channel in the electrophysiology of PFs suggests its potential role in cardiac conduction and ventricular arrhythmias.

## 4. Involvement of TRPM4 in Cardiac Arrhythmias under Pathophysiological Conditions

(1) Pathophysiology of the TRPM4 channel under hypertrophic and remodeling conditions

**Figure 1:**
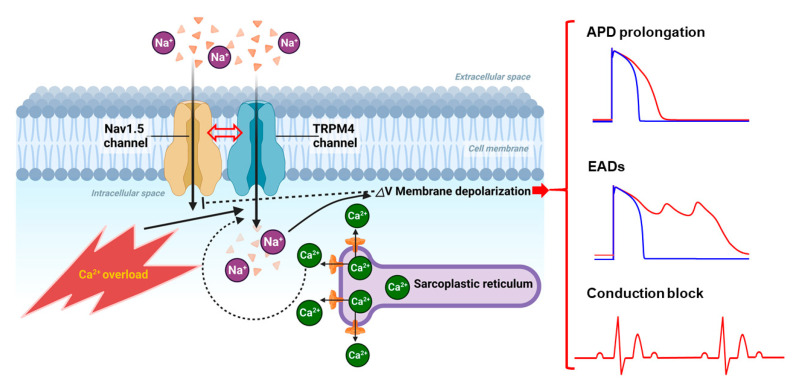
Ca^2+^ overloading conditions lead to TRPM4 overactivation inducing pleiotropic electrical abnormalities in cardiomyocytes. Under pathological conditions such as ischemia and heart failure, Ca^2+^ overload occurs with spontaneous Ca^2+^ release events [40], leading to the overactivation of TRPM4 channels. This may result in increased Na^+^ influx through TRPM4 channels particularly during the late phase of AP or even after AP termination, which in turn causes AP prolongation as well as a depolarizing shift of the resting membrane potential. When this is combined with genetic mutations and/or tissue remodeling, both of which could facilitate TRPM4 overactivation, early after-depolarizations (EADs) can be triggered, or reduction of the conduction velocity or conduction block can ensue, due to substantial inactivation of Nav1.5 channels. Moreover, there is evidence for a functional coupling between TRPM4 and Nav1.5 channels [41], which may further complicate the consequences of TRPM4 overactivation. (More details are provided in the text).

Both *Trpm4* mRNA expression and TRPM4-like currents were found to be increased in hypertrophied ventricular cardiomyocytes from SHRs, compared to normotensive WKY rats [4]. These changes were also accompanied by abnormal prolongation of QT intervals. Thus, the authors speculated that, in hypertrophied hearts, overactivation of TRPM4 channels may act as a proarrhythmic substrate for early and delayed depolarizations. Subsequent studies employing genetically engineered TRPM4-transgenic mice and numerical simulations confirmed this possibility [13,35,36,37]. Intriguingly, using the same cardiomyocyte-specific *Trpm4 KO* mice [29], TRPM4 channels were shown to act differently as either negative or positive regulators for angiotensin II-induced cardiac hypertrophy [29] and pressure overload-induced cardiac hypertrophy [38]. At present, the reason for this discrepancy is unclear and awaits further investigation. It is likely that compromised Ca^2+^ homeostasis and Ca^2+^ overload, two prominent features of remodeling and stressed hearts, facilitate TRPM4 overactivation and concurrent arrhythmias (Figure 1). The TRPM4 channel specifically contributes to Ca^2+^ overload-induced background current that enhances ectopic excitability, and its nonspecific blocker, meclofenamate, significantly suppresses catecholaminergic polymorphic ventricular tachycardia (CPVT)-associated arrhythmias at doses appropriate for TRPM4 channel inhibition [39].

Increased expression and activation of the TRPM4 channel could lead to higher susceptibility to hypertrophy- and stress-induced ventricular arrhythmias in association with aberrant Ca^2+^ homeostasis [42]. TRPM7, albeit much less abundant in the atrium than TRPM4, is significantly upregulated in human atrial myocytes isolated from the patients with atrial fibrillation [43]. Mice subjected to subcutaneous aldosterone infusion and high-salt diet underwent proarrhythmic changes in the atrium, such as enhanced triggered arrhythmias (EAD, DAD) and shortened AP, both of which were eliminated by ablation of the *Trpm4* gene. In contrast, complex morphologic changes occurred due to the *Trpm4* gene ablation, specifically, dilation of the left atrium and thickening of the septum and ventricular posterior wall. Intriguingly, only the latter two of these changes were abolished by aldosterone and high-salt treatment. The basal heart rate was insensitive to aldosterone and high salt in normal mice, but was responsive to it in the *Trpm4 KO* mice [24]. Although the mechanisms underlying remain unclear, the above findings strongly favor the vital participation of TRPM4 channels in electrical and remodeling.

(2) Role of TRPM4 in ischemia-reperfusion-related arrhythmias

Reperfusion of an ischemic heart could cause severe additional damage to the myocardium and induce lethal arrhythmias [44]. Excessive production of reactive oxygen species (ROS) and intracellular Ca^2+^ overload during ischemia-reperfusion play crucial roles in the genesis of both tissue injury and arrhythmias [45]. It has been reported that in the Langendorff ischemia-reperfusion model of rat hearts pretreated with the TRPM4 channel blocker 9-phenanthrol significantly improved contractile function and limited the infarcted area [46]. Furthermore, TRPM4 channel inhibition and silencing greatly suppressed ROS-induced injury on H9C2 cardiomyocytes [47]. The destructive contribution of TRPM4 to ROS-induced cardiac injury is potentially linked to the reduction of mitochondrial membrane potential and intracellular ATP level [48]. During ischemia-reperfusion, the electrical disturbances from PFs, where the TRPM4 channel is most abundantly and functionally expressed [32], account for the occurrence of ventricular arrhythmias [49]. The first report on the anti-arrhythmic effect of the TRPM4 channel-selective blocker 9-phenanthrol was from the treatment of arrhythmias induced by hypoxia and re-oxygenation in murine ventricles: perfusion with 9-phenanthrol demonstrated a spectacular dose-dependent abolishment of EADs [5]. Interestingly, an in vivo study of a mouse model, where acute ischemia was induced by ligation of the left anterior descending (LAD) coronary artery for 30 min, showed that *Trpm4 KO* mice are much less likely to develop ischemia-induced arrhythmias. In contrast, no significant difference in arrhythmic responses between *Trpm4 KO* mice and WT mice was observed after effective reperfusion [39].

## 5. TRPM4 Channelopathy in Inherited Cardiac Arrhythmias

Inherited cardiac arrhythmias are a group of genetic disorders that affect the electrical activity of the heart, to cause abnormal heart rhythms and potentially life-threatening arrhythmias. These disorders could be caused by mutations in the genes encoding ion channels that are responsible for controlling the flow of ions across the cell membrane and maintaining the normal rhythm of the heart [50]. Different kinds of inherited cardiac arrhythmias, including conduction blocks, Brugada syndrome (BrS), and long QT syndrome (LQTS), have been linked to dozens of *Trpm4* gene mutations, as evidenced by genetic linkage analyses and subsequent cohort studies (Table 1) [2,51,52]. It was often found that the same genotype variant of TRPM4 produces multiple phenotypes of arrhythmias while its multiple genotype mutations cause an arrhythmia with similar clinical features. The exact mechanism(s) underlying this redundancy is not yet fully understood.

(1) TRPM4 variants in cardiac conduction block

The TRPM4-p.E7K mutant was the first to be identified as a point mutation of the TRPM4 channel in a few pedigrees of patient families that manifest progressive familial conduction block type I (PFHB I) [31]. Due to impaired deregulation of the small ubiquitin modifier conjunction (SUMOylation), the TRPM4-p.E7K mutant exhibited a GOF effect, associated with greater current density and increased protein expression at the plasma membrane [31]. It has also been reported that enhanced PIP_2_ affinity and altered channel kinetics can facilitate its activation, especially around the resting membrane potential, presumably contributive to brady-arrhythmogenicity [53,54]. Several other GOF mutations of the TRM4 channel (p.R164W, p.A432T, and p.G844D) have been discovered in patient families suffering from heart block, which also involves with the same mechanism of reduced SUMOylation [3]. In contrast, electrophysiological kinetic analysis of the TRPM4-p.A432T mutant suggested that a four-fold slower deactivation, rather than excessive cell surface expression, accounted for the augmented membrane current [64]. Using a combination of electrophysiology, rapid treatments of intracellular Ca^2+^ with UV-flash photolysis, and molecular docking analysis, this research group further investigated another GOF mutant, TRPM4-p.K914R, identified from two patients with heart disease in the same family [63]. TRPM4-p.K914R demonstrated slower activation and deactivation kinetics leading to increased membrane currents. Furthermore, the 914th lysine residue was crucial as the nanoscopic interface between the S4-S5 linker, the MHR, and TRP-domain, which determines TRPM4’s functional behavior [63].

The majority of TRPM4 variations related to familial conduction block are, thus far, GOF phenotypes. However, several loss-of-function (LOF) mutations of TRPM4 are also identified in inherited cardiac conduction defects [57,60]. The mechanism underlying such complex genotype-to-phenotype relationships is unclear. For instance, a LOF mutation, TRPM4-p.T286T, was found in patients with ventricular noncompaction and cardiac conduction disease, which eventually requires implantation of a permanent pacemaker [60]. This study revealed that inhibition of TRPM4 channel activity by its selective blocker 9-phenanthrol in human induced pluripotent stem cell–derived cardiomyocytes (hiPSC-CMs) resulted in decreased mRNA levels of HEY2, TBX5, and NKX2-5, transcription factors that play important roles in postnatal conduction system maturation [60,65]. This result is consistent with that of a gene invalidation study: *Trpm4 KO* mice displayed Luciani–Wenckebach atrioventricular blocks [66]. Taken together, the above lines of evidence support the view that downregulated function of the TRPM4 channel in immature hearts could influence the development of the conduction system and myocardial structures.

(2) TRPM4 variants in BrS

BrS is an inherited cardiac disease associated with a significant risk of lethal arrhythmias that lead to sudden death [67]. About 25% of BrS patients have the LOF mutations in the SCN5A gene that encode the α-subunit of the cardiac voltage-dependent sodium channel (Nav1.5) [68]. Among the other genes, TRPM4 variants explain 2.7% to 6% of total BrS cases [52,62]. However, the exact pathogenic mechanism by which both LOF and GOF mutations of TRPM4 can lead to BrS remain elusive [52].

By systematically analyzing the genotype-to-phenotype relationship of TRPM4 mutation for BrS, new evidence was found of LOF mutations of TRPM4, especially in a heterozygous status, that may not be sufficient to generate BrS [62]. However, an important physical interaction between TRPM4 and Nav1.5 was revealed: a significant decrease in the Nav1.5 current was observed in cardiomyocytes from *Trpm4 KO* mice [41]. Further studies are needed to understand how such interactions modulate cardiac electrical activity and the significance of the interactions in the pathogenesis of BrS.

(3) TRPM4 variants in LQTS

LQTS is a congenital and arrhythmogenic ion channel disorder characterized by QT prolongation in the electrocardiogram (ECG) [69]. It is potentially life-threatening because of delayed ventricular repolarization. Mutations in three major LQTS genes (KCNQ1, KCNH2, and SCN5A) were shown to impair the AP repolarization and contribute to approximately 75% of the disorder [70]. TRPM4 variants were also found to be involved in approximately 2% of LQTS cases [51], and two of these variants, p.R499W and p.V441M, were shown to display smaller TRPM4-mediated currents compared to those in wild type [51]. There is however a caveat against simply interpreting this result as the direct cause for abnormal delayed AP repolarization. It is suggested that the role of TRPM4 variants on QT interval might be multifactorial [51], as they may modulate other membrane currents as well. It is also possible that the impact of TRPM4 mutations may only become apparent in concert with the dysfunction of other AP formation channels such as KCNQ1, KCNJ2, and CACNA1C. These channels are known to alter the QT interval via a more complex mechanism than directly affecting the AP duration. For instance, a heterozygous KCNQ1/TRPM4 dual mutation has been found in a LQTS patient, in whom verapamil treatment successfully reduced defibrillator discharge frequency by shortening the QT interval [71]. However, the underlying mechanism in the contribution of TRPM4 variants to LQTS still remains unclear.

## 6. Conclusions and Perspective

TRPM4 has recently emerged as a new factor in cardiac arrhythmogenesis of both acquired and inherited types. More than thirty single mutations in the *TRPM4* gene are now connected to risky arrhythmic manifestations, and functional upregulation/dysregulation of TRPM4 channels with ECG abnormalities is reported to be involved in pathological cardiac remodeling (in heart failure and other hypertrophic cardiomyopathies). To fully understand such multiplicity and complexity and to develop effective therapeutic interventions against them, mechanistically focused studies, that delve deeper than studies of genotype-phenotype associations, are needed. In this regard, there are several challenging but promising directions. For example, transgenic animal models can be more avidly combined with optogenetic approaches to deepen our tissue-level knowledge, in vivo or ex vivo [72,73]. The single nuclei RNA sequencing can be utilized to acquire more direct information about the geometrical heterogeneity of TRPM4 and the other arrhythmic substrates across the whole heart tissue [74], in which, appropriate heterocellular cardiac organoid models could be fabricated to disentangle the tissue-level complexity in more depth [75]. Continuous monitoring of genetically at-risk individuals, using wearable detection devices, may facilitate the uncovering of otherwise unknown triggers for TRPM4-associated arrhythmias [76]. Atomic-level structural analysis will also increase detailed knowledge of processes involved in the altered gating of the TRPM4 channel which predisposes individuals to arrhythmias [77]. All these lines of quantitative information could be integrated into multi-scale, multi-hierarchical numerical models to simulate the 2- or 3-dimensional propagation of excitation waves and their disturbances (reentries, blocks) in a more precise fashion, ultimately providing a useful tool for risk evaluation and in silico design of treatments.

The pathophysiological impact of TRPM4 dysfunction is further complicated by its tight link with [Ca^2+^]_i_ dynamics and possible modification of the other types of cardiac ion channels. Although the primary consequence of TRPM4 channel activation is presumed to prolong AP duration or QT intervals, it is also known that genetic deletion of this channel induces pleiotropic changes in the ECG [66]. In this respect, the latest finding that TRPM4 interacts with Nav1.5 [41] might offer an additional clue to deciphering why a single mutation of the *TRPM4* gene results in multiple phenotypes and vice versa, as observed in LQTS and BrS.

In preclinical studies using transgenic mice, a number of selective TRPM4 blockers have been shown to suppress arrhythmias [5,13,39,78], suggesting that targeting this channel may serve as a new therapeutic strategy. In most investigations, however, only low concentrations of 9-phenanthrol inhibited TRPM4 channels relatively selectively. Intriguingly, recent investigations identified two promising aryloxyacyl-anthranilic acid compounds, NBA and CBA, as novel TRPM4 antagonists. In particular, CBA exhibited a greater potency than 9-phenanthrol in inhibiting human TRPM4; however, had no effect on its mouse homologue [79]. These unique features may serve as a good starting point to design a new generation of TRPM4 blockers with better pharmacological profiles.

In future studies, advancements in gene editing technologies like CRISPR-Cas9 may enable direct modification of the TRPM4 gene in cells, tissues or organs. Gene editing could be utilized to correct deleterious mutations associated with TRPM4-related arrhythmias or to prevent the overactivation of TRPM4 channels in cardiac tissues undergoing pathological remodeling, which would otherwise increase the arrhythmogenicity.

Obviously, further detailed investigations about TRPM4 physiology and pathophysiology are needed to substantiate the utility of the above-mentioned therapeutic means and elucidate their true clinical benefits for arrhythmia treatments.

## Figures and Tables

**Table 1:** TRPM4 channelopathy associated with cardiac arrhythmias.

Amino Acid Alteration	Effect	Arrhythmia Phenotype	Underlying Mechanism	Reference
p.E7K	GOF	PFHBI	Impaired SUMOylation and increased surface expression/altered PIP_2_ interaction	[31,53,54,55]
p.C20S	LOF	SUD	Decreased protein expression	[56]
p.A101T	LOF	CHB	Increased degradation rate/decreased protein expression	[57]
p.A101T/P1204L	LOF	IVF	Increased degradation rate/decreased protein expression	[57]
p.Q131H	Not determined	RBBB	Not determined	[58]
p.R144W	Not determined	BrS	Not determined	[52]
p.T160M	Not determined	LQTS	Involved with KCNQ1 mutation	[59]
p.R164W	GOF	RBBB	Impaired SUMOylation and increased surface expression	[3]
p.D198G	No effect	AVB	Not determined	[55]
p.T286T	LOF	PFHBI	Failure to produce functional protein	[60]
p.Q293R	Not determined	AVB	Not determined	[58]
p.I376T	GOF	PFHBI	Increased surface expression	[61]
p.A380V	LOF	SUD	Decreased surface expression/rapid desensitization	[56]
p.A432T	GOF/LOF	AVB/RBBB/BrS	Decreased surface expression or impaired SUMOylation and increased surface expression/delayed deactivation	[3,52,55,58]
p.A432T/G582S	LOF	AVB	Decreased surface expression	[55]
p.V441M	LOF	LQTS	Not determined	[51]
p.R499W	LOF	LQTS	Not determined	[51]
p.R499P	Not determined	LQTS	Not determined	[51]
p.W525X	LOF	BrS	absence of functional protein	[62]
p.G555R	Not determined	BrS	Not determined	[52]
p.G582S	GOF	AVB/RBBB/BrS	increased surface expression	[52,55,58]
p.L595V	LOF	SUD	Decreased surface expression	[56]
p.T677I	No effect	AVB	Not determined	[55]
p.G737R	Not determined	BrS	Not determined	[52]
p.F773I	Not determined	BrS	Not determined	[52]
p.P779R	LOF	BrS	Decreased protein expression	[52]
p.Y790H	Not determined	AVB	Not determined	[58]
p.G844D	GOF	LQTS/RBBB/BrS	Impaired SUMOylation and increased surface expression	[3,51,52]
p.Q854R	GOF	RBBB/BrS	Reduced degradation rate/increased protein expression	[52,57,58]
p.T873I	No effect	BrS	increased surface expression	[52]
p.K914R	GOF	AVB	Delayed deactivation	[58,63]
p.K914X	LOF	BrS	Decreased protein expression/decreased surface expression	[52]
p.V921I	No effect	AVB	Not determined	[55]
p.P970S	Not determined	RBBB	Not determined	[58]
p.S1044C	LOF	CHB	Increased degradation rate/decreased protein expression	[57]
p.L1075P	No effect	BrS	Increased surface expression	[52]
p.I1082S	LOF	SUD	Not determined	[56]
p.P1204L	Not determined	BrS	Not determined	[58]

PFHB1: progressive familial heart block type I; SUD: sudden unexpected death; CHB: complete heart block; IVF: idiopathic ventricular fibrillation; RBBB: right bundle branch block; BrS: Brugada syndrome; LQTS: long QT syndrome; AVB: atrioventricular block.

## Data Availability

The research data described in this paper are available on request.
